# Developing leadership in psychiatry

**DOI:** 10.1192/j.eurpsy.2026.10260

**Published:** 2026-07-31

**Authors:** M. Az

**Affiliations:** Amazon Spain Services, S.L.U., Barcelona, Spain

## Abstract

**Abstract:**

Leadership in psychiatry is gaining increasing importance as healthcare systems grow more dynamic and complex. Despite psychiatrists’ essential role in mental health care, structured leadership education during medical school and residency remains insufficient. As a result, many clinicians develop leadership abilities through practical experience rather than formal training. This presentation emphasizes the need to make leadership development a standard and mandatory component of psychiatric education. Clinical leadership in mental health is not only about running services efficiently. Management focuses on keeping systems organized and using resources properly, but leadership is about setting a clear path and helping others move in the same direction. A psychiatrist in a leadership role needs clinical knowledge as well as strong people skills, ethical values, and the ability to see the bigger picture. They are expected to organize services, guide teams, use resources wisely, maintain high professional standards, and respond to wider system challenges. Leadership also involves leading change, encouraging others, and balancing the needs of individual patients with the needs of the community as a whole. Some aspects of leadership may be natural, but many key abilities such as clear communication, leading teams, and thinking ahead can be taught and strengthened through structured education and guidance. Making leadership training part of psychiatric residency programs can contribute to stronger mental health services, better patient outcomes, and the ongoing development of psychiatry in a rapidly changing healthcare environment.

**Disclosure of Interest:**

None Declared

